# Recognising Emotions from the Voice: A tDCS and fNIRS Double-Blind Study on the Role of the Cerebellum in Emotional Prosody

**DOI:** 10.3390/brainsci15121327

**Published:** 2025-12-13

**Authors:** Sharon Mara Luciano, Laura Sagliano, Alessia Salzillo, Luigi Trojano, Francesco Panico

**Affiliations:** Department of Psychology, University of Campania “Luigi Vanvitelli”, Viale Ellittico 31, 81100 Caserta, Italy; sharonmara.luciano@unicampania.it (S.M.L.); alessia.salzillo1@unicampania.it (A.S.); luigi.trojano@unicampania.it (L.T.); francesco.panico@unicampania.it (F.P.)

**Keywords:** cerebellum, prefrontal cortex, emotional prosody, emotions, transcranial direct current stimulation, functional near-infrared spectroscopy

## Abstract

**Background**: Emotional prosody refers to the variations in pitch, pause, melody, rhythm, and stress of pronunciation conveying emotional meaning during speech. Although several studies demonstrated that the cerebellum is involved in the network subserving recognition of emotional facial expressions, there is only preliminary evidence suggesting its possible contribution to recognising emotional prosody by modulating the activity of cerebello-prefrontal circuits. The present study aims to further explore the role of the left and right cerebellum in the recognition of emotional prosody in a sample of healthy individuals who were required to identify emotions (happiness, anger, sadness, surprise, disgust, and neutral) from vocal stimuli selected from a validated database (EMOVO corpus). **Methods**: Anodal transcranial Direct Current Stimulation (tDCS) was used in offline mode to modulate cerebellar activity before the emotional prosody recognition task, and functional near-infrared spectroscopy (fNIRS) was used to monitor stimulation-related changes in oxy- and deoxy- haemoglobin (O2HB and HHB) in prefrontal areas (PFC). **Results**: Right cerebellar stimulation reduced reaction times in the recognition of all emotions (except neutral and disgust) as compared to both the sham and left cerebellar stimulation, while accuracy was not affected by the stimulation. Haemodynamic data revealed that right cerebellar stimulation reduced O2HB and increased HHB in the PFC bilaterally relative to the other stimulation conditions. **Conclusions**: These findings are consistent with the involvement of the right cerebellum in modulating emotional processing and in regulating cerebello-prefrontal circuits.

## 1. Introduction

The term prosody refers to the component of speech conveying various shades of meaning through modulations of accent and tone, independently from grammatical structure and lexical content [1]. Many authors [2,3,4] distinguish between the linguistic functions of prosody (e.g., differentiating a statement from a question) and its non-linguistic functions, which enable the listener to infer the speaker’s emotional state and communicative intent even in the absence of explicit semantic information. The ability to encode and decode emotional prosody is central to social communication and requires the integration of auditory, emotional, and cognitive processing mechanisms [5]. Functional neuroimaging studies have traditionally identified a fronto-temporal network, primarily right-lateralised, as essential to the processing of emotional prosody [6,7]. However, recent empirical data have put forward the possible contribution of the cerebellum in such processing, as cerebellar stimulation reduced neural activation in the prefrontal cortex during an emotional prosody recognition task [8], and lesions in the right cerebellum (Lobules VIIb and VIII and right Crus I and II) have been associated with impairments in both the sensory and cognitive processing of emotions [9,10].

Early anatomical studies conducted in animal models revealed bidirectional pathways linking the cerebellum to major cortical areas relevant for many cognitive and emotional functions [10]. These studies described robust cerebello-prefrontal connectivity pathways, where the prefrontal cortex (PFC) projects to the contralateral cerebellum via the pontine nuclei, and conversely, the cerebellum sends projections back to the contralateral PFC via the dentate nucleus and thalamus [11]. The cerebellum is also connected to subcortical structures involved in emotion processing, such as the amygdala and the basal ganglia [12,13]. More recently, neuroimaging studies in humans have identified specific regions of the posterior cerebellum, including lobules IV–IX and Crus I/II, implicated in emotional prosody recognition [14], and specific cerebello-frontal loops involving the thalamus and prefrontal cortex [15]. This evidence aligns with clinical findings from patients with cerebellar stroke, who often misidentify emotions when lesions affect the right hemisphere [9]. Further support for a lateralised functional organisation of the cerebellum in emotional processing comes from studies on Parkinson’s disease [16], even though a predominant involvement of the right cerebral hemisphere has been supported by some studies [17], but not others [18,19].

Non-invasive brain stimulation techniques have emerged as promising tools for causally probing the role of the cerebellum and of its connections with cortical regions in cognitive and emotional functions [20]. Specifically, transcranial direct current stimulation (tDCS) has been shown to selectively modulate cerebellar activity, producing measurable effects on behaviour and on the activity of distant brain regions, such as the PFC [21]. Moreover, tDCS can be coupled with portable neuroimaging methods, such as functional near-infrared spectroscopy (fNIRS) [8], allowing a powerful integration for investigating cerebellar influence on higher-order functional circuits in ecologically valid and non-constrained settings.

While cerebellar tDCS has been already shown to modulate performance in visual emotion recognition tasks, especially by reducing reaction times (RTs) for negative facial expressions after both anodal and cathodal (but not sham) stimulation [22], its effects on auditory emotional processing remain largely underexplored. A recent study by Panico et al. [8], employing an *online* stimulation protocol, investigated the impact of cerebellar stimulation on a emotion prosody recognition task and related PFC activity patterns. This study supported the concept of a cerebello-prefrontal task-related functional network, whose modulation, however, had no measurable effect on behavioural performance, thus leaving unresolved the specific contribution of the cerebellum to this kind of task. As a matter of fact, the literature on the effects of cerebellar tDCS reports promising but variable results, largely dependent on parameters such as intensity, polarity, electrode montage, duration, and crucially, timing of stimulation (online vs. offline modes; [23]). For instance, studies in healthy older adults have shown improvements in motor learning only 48 h after a session of cerebellar anodal tDCS, supporting a selective effect on offline learning rather than during task execution [24]. Similarly, in patients with Parkinson’s disease, an offline stimulation protocol enhanced recognition of sadness, suggesting that stimulation applied outside the task may promote more stable consolidation processes [22].

Considering these latter studies supporting the use of offline designs in cerebellar stimulation, the present study had a twofold aim. The first aim was to examine whether *offline* tDCS [22] can affect behavioural performance during an emotional prosody recognition task in healthy individuals. This would provide fresh evidence on the effective contribution of the cerebellum, and its possible lateralisation, in emotional prosody recognition. The second aim was to assess whether cerebellar stimulation can modulate PFC activity, measured via fNIRS, before and after the stimulation.

Building on previous evidence on the involvement of the cerebellum in facial emotion recognition [22] and on the growing body of work highlighting the cerebellar contribution to socio-cognitive and affective functions [16,25], we expected cerebellar stimulation to modulate emotional prosody recognition performance. In line with previous findings on auditory emotion recognition, we also anticipated that recognition performance would vary across emotions, with negative emotions being affected more than neutral and positive ones [8,26]. Moreover, as cerebellar tDCS has been shown to influence the PFC activity during emotional prosody recognition [8], and neuroimaging and lesion studies supported the cerebellar involvement in the perception and integration of emotional prosody via connections with cortical regions such as the PFC [4,27], we hypothesised that modulating cerebellar excitability through tDCS would not only influence the accuracy and RTs during prosody decoding, but it would also alter PFC activation patterns as measured by fNIRS, consistent with existing models of cerebello-frontal functional connectivity [28].

## 2. Materials and Methods

### 2.1. Participants and Experimental Procedure

The study employed a double-blind, within-subject design. An a priori power analysis was conducted using G*Power 3.1 [29] based on a medium effect size (Cohen’ s f) and standard parameters in the absence of robust, task-specific estimations to establish more precise assumptions. This analysis provided a minimum total sample size of 14 participants to conduct repeated-measures ANOVAs with two within-subject factors (emotion: 6 levels; stimulation: 3 levels; see thereafter) and detect an effect size of 0.25, with a power of 0.95 and an alpha of 0.05. Eighteen right-handed university students (8 male; age range: 20–31 years; mean age: 24.6, SD = 3.6) voluntarily participated in a within-subject, double-blinded stimulation study involving three experimental sessions. Upon enrolment, the participants underwent a screening interview to confirm eligibility for stimulation and provided their written informed consent. Participants who reported normal or corrected-to-normal vision, normal hearing, and no history of neurological or psychiatric disorders were included in the study. Participants were naïve to the aims of the experiment.

During each session, the participants received tDCS before performing a voice emotion recognition task (offline mode). Prefrontal brain activity was recorded using a wearable fNIRS device before and after stimulation (see below). The study protocol and procedures adhered to the principles of the 1975 Declaration of Helsinki and received ethical approval from the Ethics Committee of the Department of Psychology (protocol no. 21/2023).

### 2.2. Vocal Emotion Recognition Task

The vocal emotion recognition task was developed using the PsyToolkit platform version 3.4.1 [30]. As in Panico et al. [8], the participants listened to audio recordings selected from a validated Italian database (EMOVO corpus; [26]), in which professional actors (two male and two female actors) utter four meaningless sentences with varying emotional intonations (happiness, anger, sadness, surprise, disgust, and neutral). The task included a total of 96 stimuli (4 sentences × 4 speakers × 6 emotions), presented in two pseudorandomised blocks of 48 stimuli each plus a brief training session consisting of 16 practice trials to allow participants to become familiar with the task structure; each audio stimulus lasted 3 s. Audio files were delivered binaurally through stereo headphones, with a fixed volume level of 65% of maximum PC output for all participants. A one-minute break was introduced between blocks to reduce fatigue. The set of stimuli was held constant across the three experimental sessions, but the order of presentation was randomised anew in each session.

The participants were required to identify the emotion conveyed by the voice by pressing a number key (1 to 6; counterbalanced across participants) corresponding to the six possible emotional categories. Responses were possible only after the audio had finished playing, and the next trial was presented immediately after participants’ response. The maximum response time was set at 5000 ms; the inter-stimulus interval was set at 500 ms. No feedback on accuracy was provided during the task. The full task duration was approximately 18 min.

### 2.3. Transcranial Direct Current Stimulation (tDCS)

tDCS was administered using an offline protocol mode, before the emotional recognition task, according to previous studies showing that the main effects of cerebellar stimulation can outlast the stimulation period [22]. A constant current of 2.0 mA was delivered via a battery-powered stimulator (BrainSTIM, EMS Medical, Bologna Italy), through two saline-soaked sponge electrodes (5 cm × 5 cm; surface area = 25 cm^2^). The anodal electrode was positioned over either the right or left cerebellar hemisphere (anodal r-Cb or anodal l-Cb), with the centre of the electrode placed 3 cm lateral to the inion, while the cathodal electrode was placed over the ipsilateral buccinator muscle [31]. This monocephalic montage was selected to minimise the potential influence of tDCS on cortical areas beyond the cerebellum [8], as confirmed by computational modelling indicating electric field spreading to the lateral posterior cerebellar regions [32]. In the sham condition (sham tDCS), electrode placement was identical to the active stimulation, but the current was discontinued after the initial 30 s. This approach was used to reproduce the tingling sensation typically experienced at stimulation onset, thereby maintaining participant blinding across conditions [33]. The position of the anodal electrode in the sham condition was counterbalanced across participants and placed on the right cerebellum in half of the participants and on the left cerebellar in the other half. The stimulation duration was set at 21 min in total (see [8,22]), including 30 s ramp-up and ramp-down periods, in accordance with established safety guidelines for tDCS [34]. The order of stimulation was determined by an experimenter (LS) not involved in data collection on a predefined randomisation sequence to ensure double-blinding; each stimulation session was spaced one-week apart.

### 2.4. Functional Near-Infrared Spectroscopy (fNIRS)

To measure the PFC activity of regions implicated in cerebello-prefrontal connectivity [35,36], we employed a wearable fNIRS system (OctaMon, Artinis Medical Systems, Elst, The Netherlands) that recorded oxygenated (O2HB) and deoxygenated (HHB) haemoglobin in the resting state before and after each stimulation session for 5 min [8,37]. The fNIRS system operated at two wavelengths (758 nm and 840 nm) to detect variations in light absorption; the differential pathlength factor (DPF) was adjusted individually based on participant age, following established procedures [38]. Concentrations of O2HB and HHB (expressed in ΔμM) were calculated using the modified Beer–Lambert law. The optical setup included eight LED emitters (four per hemisphere) and two photodetectors (one per hemisphere), embedded in a flexible probe holder, and spaced 35 mm apart, enabling the formation of eight measurement channels: channels 1–4 covered the right hemisphere and channels 5–8 the left hemisphere. Detectors were aligned over FP1 and FP2 positions of the 10–20 international EEG system [8,39]. Throughout the recording, optode-skin contact quality was continuously monitored. Real-time data visualisation and acquisition were managed through OxySoft software version 3.2.51.4 (Artinis Medical Systems, Elst, The Netherlands), with a sampling frequency of 10 Hz.

### 2.5. Data Analysis

Based on the performance at the vocal emotion recognition task, a minimum overall accuracy of 60% was set a priori as an inclusion criterion, and participants who did not reach this threshold were planned to be excluded from the analyses. Normality of quantitative variables was assessed using skewness and kurtosis indices, with values within the range of −2–2 deemed indicative of approximate normal distributions [40]. Raw data were transformed into Z-scores separately for each participant. Outliers were defined as values with z > |3| and, when present, were replaced with the corresponding group median for that participant to limit the impact of extreme scores while preserving the within-subject structure of the data [41,42].

Behavioural data were analysed by running separate 3 × 6 repeated-measures ANOVAs on accuracy (calculated as the percentage of correct responses) and RTs (calculated as the averaged response time on correct responses), with Stimulation (anodal r-Cb, vs. anodal l-Cb, vs. sham tDCS) and Emotion (neutral, happiness, disgust, anger, sadness, surprise) as within-subject factors.

For the haemodynamic data, we assessed the signal quality and the absence of motion artefacts through visual inspection [43]. The fNIRS data were pre-processed in OxySoft using a Moving Gaussian filter with a filter width set at 1 which is designed to attenuate physiological noise, also including motion-related artefacts [43,44]. Data recorded in the eight channels (Ch 1–4 and Ch 5–8) were averaged in two regions of interests (ROIs) corresponding, respectively, to the right and left PFC. To quantify changes in the PFC activity following cerebellar stimulation, a neural activation variation index was computed by subtracting pre-stimulation values from post-stimulation values (Δ = Post-Pre, [8]), with positive values indicating increased neural activation following stimulation.

In case of significant interaction effects, Bonferroni adjusted post hoc pairwise comparisons were performed to control for type I error. A *p*-value < 0.05 was considered statistically significant. Effect sizes were estimated using eta-squared (η^2^) statistics.

Statistical analyses were performed by using IBM SPSS Statistics v. 20 and Jamovi v. 2.6.44.

## 3. Results

### 3.1. Behavioural Results: Accuracy

All participants met the pre-established accuracy threshold (≥60%), therefore no participant was excluded from the analyses based on this criterion. The repeated-measures ANOVAs on accuracy showed a significant main effect of Emotion (F(5, 85) = 51.763, *p* < 0.001, η^2^ = 0.571), while neither the factor Stimulation (F(2, 34) = 0.064, *p* = 0.938) nor the interaction between Stimulation and Emotion (F(10, 170) = 1.043, *p* = 0.41) were significant. Post hoc comparisons showed that participants were less accurate in recognising disgust as compared to neutral (*p* = 0.001), anger (*p* < 0.001), and sadness (*p* < 0.001). Moreover, the recognition of happiness was less accurate than recognition of neutral (*p* < 0.001), anger (*p* < 0.001), and sadness (*p* = 0.001), but more accurate than the recognition of surprise (*p* = 0.01). Neutral was recognised more accurately than surprise (*p* < 0.001). Similarly, anger was recognised more accurately than surprise (*p* < 0.001) and sadness (*p* = 0.047). Finally, surprise was recognised less accurately than sadness (*p* < 0.001) (Table 1).

### 3.2. Behavioural Results: Reaction Times

Seven outlier values (z > |3|) were replaced with the median of their respective condition. Repeated-measures ANOVA on RTs showed a significant main effect of Stimulation (F(2, 32) = 59.64, *p* < 0.001, η^2^ = 0.285), as a significant reduction in RTs was observed following r-Cb (*M* = 414 ± 12.0) compared to the sham (*M* = 861 ± 54.1, *p* < 0.001) and l-Cb (*M* = 730 ± 44.7, *p* < 0.001), and reduced RTs following l-Cb compared to the sham (*p* < 0.001) and a significant main effect of Emotion (F(5, 80) = 8.54, *p* < 0.001, η^2^ = 0.063), showing longer RTs in identifying disgust (*M* = 820 ± 73.3) compared to neutral (*M* = 633 ± 47.4, *p* = 0.015), surprise (*M* = 548 ± 35.8, *p* = 0.015), and sadness (*M* = 600 ± 35.6, *p* = 0.015), longer RTs when identifying happiness (*M* = 690 ± 38.5) compared to surprise (*p* = 0.017), and finally, longer RTs in recognising anger (*M* = 720 ± 19.3) compared to surprise (*p* = 0.001).

**Figure 1:**
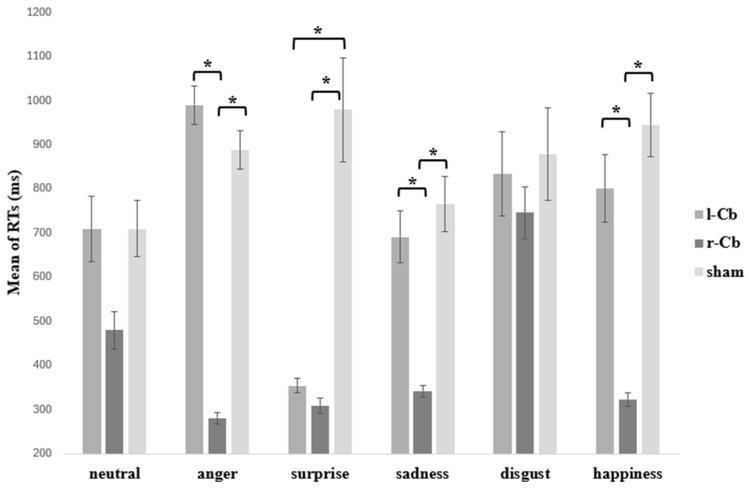
Mean RTs (ms) for correct responses to the emotional prosody recognition task for each emotion and stimulation session. Error bars represent standard errors; * = significant for *p* < 0.05 Bonferroni-corrected.

Interestingly a significant interaction between Stimulation and Emotion was found (F(10, 160) = 11.72, *p* < 0.001, η^2^ = 0.134; Figure 1). Post hoc between-stimulation contrasts by emotion showed that recognition of happiness was faster after r-Cb (*M* = 323 ± 15.3) than after sham (*M* = 944 ± 72.2, *p* < 0.001) and l-Cb (*M* = 801 ± 75.5, *p* = 0.002), with no difference between sham and l-Cb (*p* > 0.05). Recognition of anger was faster after r-Cb (*M* = 281 ± 12.9) than after sham (*M* = 888 ± 43.7, *p* < 0.001) and l-Cb (*M* = 990 ± 43.6, *p* < 0.001), with no difference between sham and l-Cb (*p* > 0.05). Recognition of surprise was faster after r-Cb (*M* = 309 ± 18.0) than sham (*M* = 979 ± 118.5, *p* = 0.007); furthermore, RTs did not differ between r-Cb and l-Cb (*M* = 354 ± 16.5, *p* > 0.05), and participants were slower after sham compared with l-Cb (*p* = 0.03). Recognition of sadness was faster after r-Cb (*M* = 342 ± 13.1) than sham (*M* = 766 ± 62.1, *p* < 0.001) and l-Cb (*M* = 691 ± 59.1, *p* = 0.005), with no difference between sham and l-Cb (*p* > 0.05). Finally, for disgust and neutral, RTs did not differ across r-Cb (Disgust: *M* = 746 ± 58.5; Neutral: *M* = 480 ± 42.6), l-Cb (Disgust: *M* = 834 ± 95.6; Neutral: *M* = 480 ± 42.6), or sham stimulation (Disgust: *M* = 879 ± 103.9; Neutral: *M* = 710 ± 64.0; all *p* > 0.05).

With regard to within-stimulation conditions, in r-Cb stimulation participants took longer to recognise disgust than happiness (*p* < 0.001), anger (*p* < 0.001), surprise (*p* < 0.001), and sadness (*p* < 0.001), with no difference relative to neutral (*p* = 0.223). RTs for happiness did not differ from neutral, anger, surprise, or sadness (all *p* > 0.05). Similarly, neutral did not differ as compared to anger (*p* = 0.059), surprise (*p* = 0.509), or sadness (*p* > 0.05); anger did not differ from surprise (*p* > 0.05) or sadness (*p* = 0.32). Moreover, in l-Cb stimulation participants took significantly longer to recognise disgust relative to surprise (*p* = 0.043), but not compared with happiness, neutral, anger, or sadness (all *p* > 0.05). Happiness was also recognised more slowly than surprise (*p* = 0.007), but RTs for happiness did not differ from those observed during neutral, anger, or sadness (all *p* > 0.05). RTs for neutral did not differ from anger (*p* > 0.05), surprise (*p* = 0.06), or sadness (*p* > 0.05). RTs for anger were larger than those recorded for surprise (*p* < 0.001), while no difference was found compared with sadness (*p* = 0.19). Sadness, in turn, was recognised more slowly than surprise (*p* = 0.008). In the sham condition, no differences were observed in RTs for disgust compared with happiness, neutral, anger, surprise, or sadness (all *p* > 0.05). Similarly, happiness did not differ from neutral, anger, surprise, or sadness (all *p* > 0.05). RTs for neutral did not differ from anger (*p* = 0.84), surprise (*p* > 0.05), or sadness (*p* > 0.05). Anger did not differ from surprise (*p* > 0.05) or sadness (*p* > 0.05), and sadness did not differ from surprise (*p* > 0.05; Figure 1).

### 3.3. Haemodynamic Data: Delta Post-Pre (Δ) O2HB

**Figure 2:**
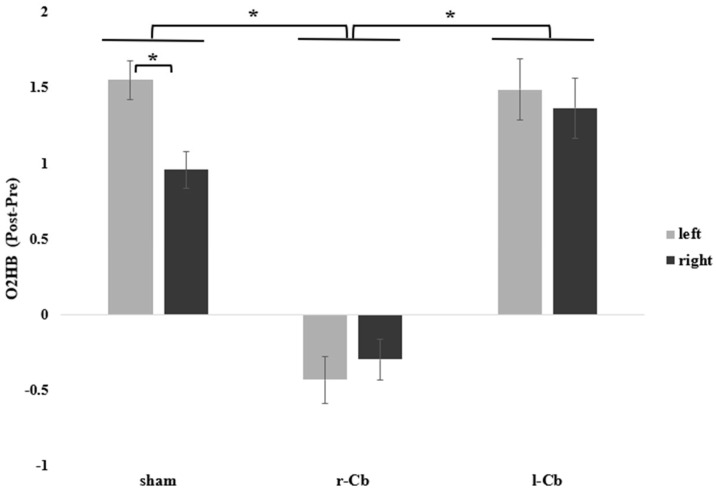
Differences in the neural activation index (Post-Pre) showing a reduction in O2HB levels in the PFC following r-Cb stimulation (as compared to sham and l-Cb stimulation) and greater O2HB levels in the left compared to the right hemisphere under sham stimulation (no hemispheric differences were observed under r-Cb or l-Cb stimulations); error bars represent standard error, * = significant for *p* < 0.05, Bonferroni corrected.

Repeated-measures ANOVA on Δ O2HB haemodynamic data revealed a significant main effect of Stimulation (F(2, 34) = 27.15, *p* < 0.001, η^2^ = 0.411), with a reduction in O2HB levels in the PFC following r-Cb (*M* = −0.364 ± 0.095) stimulation as compared to sham stimulation (*M* = 1.253 ± 0.104; *p* < 0.001) and l-Cb stimulation (*M* = 1.428 ± 0.163, *p* < 0.001); no difference was observed between l-Cb and sham stimulation (*p* = 0.75). While no main effect of Hemisphere was found (F(1, 17) = 2.20, *p* = 0.156), a significant interaction effect between Stimulation and Hemisphere (F(2, 34) = 4.15, *p* = 0.024, η^2^ = 0.020; Figure 2) was detected. Post hoc analyses revealed a significant hemispheric difference in brain activation under sham stimulation only, with greater O2HB in the left (*M* = 1.549 ± 0.127) compared with the right hemisphere (*M* = 0.957 ± 0.119, *p* = 0.004), while no hemispheric differences were observed under r-Cb or l-Cb stimulations (all *p* > 0.05).

### 3.4. Haemodynamic Data: Delta Post-Pre (Δ) HHB

**Figure 3:**
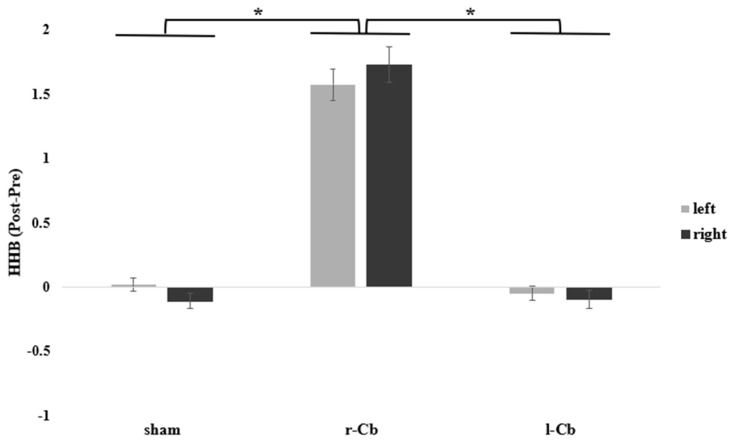
Differences in the neural activation index (Post-Pre) describing an increase in HHB levels in the left and right PFC following r-Cb stimulation as compared to sham and l-Cb stimulations; error bars represent standard error, * = significant for *p* < 0.05, Bonferroni corrected.

Repeated-measures ANOVA on Δ HHB haemodynamic data showed a significant main effect of Stimulation (F(2, 34) = 175,35 *p* < 0.001, η^2^ = 0.826), with an increase in HHB levels in the PFC following r-Cb stimulation (*M* = −0.044 ± 0.046) as compared to sham stimulation (*M* = 1.650 ± 0.092; *p* < 0.001) and l-Cb stimulation (*M* = 0.071 ± 0.056, *p* < 0.001) (Figure 3). No significant differences were observed between l-Cb stimulation and sham stimulation (*p* = 0.748). Neither a significant main effect of Hemisphere (F(1, 17) = 0.009, *p* = 0.923) nor an interaction effect was detected (F(2, 34) = 1.448, *p* = 0.249).

## 4. Discussion

The present study investigated the role of the cerebellum in emotional prosody recognition by applying an offline tDCS protocol over the right or the left cerebellar hemispheres (as compared to sham stimulation); fNIRS was used to assess possible changes in prefrontal cortical activity after tDCS stimulation.

The main findings from the present study can be summarised as follows: (i) accuracy in recognising emotions was unaffected by cerebellar stimulation; (ii) RTs were strongly modulated by stimulation, with faster responses after r-Cb stimulation with respect to both l-Cb and sham conditions; and (iii) prefrontal O2HB decreased and HHB increased following r-Cb, consistent with reduced prefrontal activation.

### 4.1. Behavioural Measures and Effects of Cerebellar Stimulation

In line with previous studies on emotional prosody recognition [8,26], participants showed variable accuracy across emotional categories. Disgust and surprise were particularly difficult to identify, whereas neutral, anger, and sadness were recognised more accurately. This pattern replicates well-documented differences in perceptual salience and acoustic distinctiveness of prosodic cues [3,45]. Importantly, no effect of cerebellar stimulation on accuracy was found, suggesting that cerebellar tDCS did not alter the ability to correctly categorise emotions in healthy individuals. Although the η^2^ values for the RTs effects were modest, results on RTs revealed a robust modulation by cerebellar stimulation. Specifically, r-Cb significantly facilitated responses to three emotions (happiness, anger, and sadness) compared with both the sham and l-Cb. There was no significant effect on the RTs for responses to disgust and neutral stimuli. Both l-Cb and r-Cb significantly facilitated responses to surprise stimuli, compared to the sham.

These findings converge with evidence from visual emotion recognition studies [22] and extend them to the auditory domain. In both the visual and auditory domains, recognition of some negative emotions (i.e., anger and sadness) was affected by cerebellar stimulation, which is compatible with a modulatory contribution of the cerebellum to affective processes. However, in the present study, and beyond our initial hypotheses, RTs for happiness and surprise were found to be significantly influenced, in contrast with findings of Ferrucci et al. [22] in the visual domain. Diverging patterns across studies, such as the absence of modulation for disgust and only modest effects for neutral stimuli in our data, may reflect differences in the nature of the stimuli. While Ferrucci’s task involved facial expressions, processed primarily through a single, non-linguistic visual channel, the task employed here relied on prosodic cues embedded in meaningless sentences. Although devoid of semantic content, these vocal stimuli still require an initial stage of linguistic processing, possibly engaging a more distributed or multi-level processing route. Taken together, these differences point to the possibility that cerebellar modulation of emotion recognition may depend on both the emotional content and the communicative modality through which emotions are conveyed.

The pattern of RTs facilitation following r-Cb stimulation is compatible with the involvement of the right cerebellar hemisphere in emotional processing [16], in line with previous evidence on hemispheric specialisation [46]. Consistently, recent studies on cerebellar tDCS modelling showed that montages similar to the one adopted here, targeting the lateral cerebellum, induce relatively high electric field strength in lobules Crus I/II, VIIb, VIII, and IX [32], which are thought to be involved in emotional processing. While not conclusive, this convergence between the behavioral pattern and the predicted stimulation profile is consistent with the idea that the montage adopted in the present study may have modulated activity in territories involved in affective processing.

### 4.2. Neural Effects and Cerebello-Prefrontal Connectivity

The fNIRS results showed a reduction in O2Hb and a concomitant increase in HHb in PFC following r-Cb stimulation compared to both the sham and l-Cb stimulations. This pattern is compatible with a decrease in prefrontal haemodynamic activity, in line with recent evidence showing that cerebellar stimulation can modulate distant cortical regions [8,21]. Interestingly, this neural modulation was observed in the absence of accuracy changes, whereas it could be related to faster RTs. One interpretation is that reduced prefrontal recruitment reflects a more efficient processing state, where less cognitive effort is required to perform the task after right cerebellar stimulation [47]. This interpretation would align with the view that the cerebellum could serve as a predictive processor optimising cortical computations [10,25]; however, the offline nature of our experimental design could not provide direct support to this hypothesis. It is also interesting to note that no hemispheric differences in prefrontal responses emerged after active stimulation, in contrast with the left-lateralised dominance observed under sham stimulation. This observation may suggest that cerebellar stimulation attenuates the typical hemispheric asymmetries of prefrontal activation during prosody recognition [9].

The present data provided further evidence that cerebellar stimulation can influence prefrontal haemodynamics, in line with the concept of cerebello–prefrontal loops whereby the cerebellum exerts an influence on cognitive and affective processes via crossed connections with contralateral prefrontal regions [48]. However, the low spatial resolution of our montage and the use of a global pre–post index computed from a limited number of prefrontal channels did not allow drawing definite conclusions about inter-hemispheric dynamics. Nonetheless, the present results would extend prior findings on facial emotion recognition, as Ferrucci et al. [22] showed that cerebellar stimulation enhances emotion decoding. Furthermore, the bilateral engagement observed here may reflect a shift from a lateralised, specialised processing strategy to a more integrated network approach. This could imply that right cerebellar stimulation modulates the distribution of neural resources required for prosody recognition, potentially balancing the activity across the two hemispheres, consistent with models of cerebello-cortical loops that emphasise bidirectional influences [11]. However, the absence of differences in participants’ accuracy between r-Cb and l-Cb, the bilateral nature of the haemodynamic changes, and the lack of direct cerebellar measures, should induce a caution in drawing conclusions about cerebellar lateralisation and PFC activity.

### 4.3. Offline Versus Online Stimulation Protocols

A key contribution of this study is the use of an offline tDCS protocol to target emotional prosody and its neural correlates. Unlike Panico et al. [8], who reported prefrontal modulation without behavioural changes using online stimulation, the present offline protocol was associated with changes in RTs together with modulation of prefrontal haemodynamic. This finding resonates with evidence from emotional recognition studies and motor learning [22,24] suggesting that cerebellar stimulation delivered offline may be followed by behavioural benefits. The present study did not include both offline and online stimulation, preventing any direct comparison between the two protocols, but stimulation timing appears to be one relevant parameter to be manipulated systematically in further experimental designs on this issue. Notably, online protocols often elicit cortical changes detectable via neuroimaging without the corresponding behavioural benefits [8,24], suggesting that the timing of stimulation is critical to influence cerebellar efferent pathways to cortical regions effectively. All in all, beyond the specific emotional domain, the current study, together with previous investigations on closely related topics [8,22], contributes to the broader literature on cerebellar tDCS by raising attention on the relevance of stimulation modality and parameters in modulating behavioural and neurofunctional responses. For instance, Ferrucci and Priori [23] emphasised that polarity and intensity can differentially modulate cerebello-cortical excitability, and can enhance cognitive or affective processing, while Grimaldi et al. [21] underlined the role of electrode montage and the current direction in shaping the specificity and spread of cerebellar tDCS effects.

### 4.4. Limitations and Future Directions

Some limitations of the present study should be acknowledged. First, tDCS has a relatively low spatial resolution, which could be particularly critical in a small and highly convoluted structure such as the cerebellum [32]. Moreover, the only use of anodal stimulation precluded the exploration of possible polarity-specific effects. Analogously, the fNIRS system used here focused on PFC only, thus not capturing potential stimulation effects on temporal or parietal regions involved in prosody processing. Future fNIRS studies should fill this gap, possibly implementing online, trial-level designs to better characterise emotion-specific prefrontal responses to vocal stimuli.

A second line of limitation is related to the task employed, as only a limited set of sentences and actors was selected, thus possibly reducing ecological validity. Furthermore, the same set of stimuli was presented across sessions. Although a counterbalanced order of stimulation conditions and a randomised trial presentation were employed with the aim of reducing learning, habituation, or memory effects, it is possible that this reduced variability. It is also worth mentioning that future studies might add a specific control for session-order effects and a formal manipulation check to ascertain effective participant blinding and exclude expectation effects.

We acknowledge that the sample size for the current study was estimated using conventional parameters due to the absence of robust, task-specific estimates [49,50]. Future research could utilise our effect size estimates to ensure optimal power.

Concerning the statistical approach, the conventional inferential statistical approach used in this study may have limited the estimation of the captured variance. Future studies specifically powered to run mixed-effects analyses [51,52] may allow capturing trial and subject variability more finely.

Finally, it would be interesting to assess emotional abilities, perceived stress, and other psychological characteristics by self-report questionnaires (not administered here) to better comprehend inter-individual variability in emotional prosody recognition and in response to cerebellar stimulation.

## 5. Conclusions

Notwithstanding its limitations, the present study provides the first evidence that offline right cerebellar anodal stimulation can enhance the speed of processing in an emotional prosody recognition task and modulate prefrontal haemodynamic responses. These findings are consistent with the involvement of the cerebellum, specifically the right cerebellar lobe, in the efficient decoding of vocal emotions, likely through its interactions with prefrontal regions. By highlighting the specific effects of offline stimulation, this study contributes to a growing understanding of cerebello-frontal circuits in socio-emotional processing and highlights the potential of cerebellar tDCS as a tool for modulating higher-order affective functions.

## Figures and Tables

**Table 1:** Accuracy at the emotional prosody recognition task for each emotion, in each stimulation condition.

Emotion	l-Cb (Mean ± SE)	r-CB (Mean ± SE)	Sham (Mean ± SE)	Total (Mean ± SE)	95% CI for the Mean CI Lower–CI Upper
Neutral	86.5 ± 2.69	84.7 ± 2.96	81.9 ± 3.5	84.4 ± 2.57	78.9–89.8
Anger	83.3 ± 2.14	84.7 ± 2.04	83.3 ± 2.67	83.8 ± 1.74	80.1–87.5
Surprise	47.2 ± 4.05	45.5 ± 2.66	48.3 ± 2.57	47.0 ± 2.51	41.7–52.3
Sadness	73.3 ± 3.07	73.6 ± 2.55	75.0 ± 3.64	74.0 ± 2.51	68.7–79.2
Disgust	54.9 ± 3.41	54.5 ± 2.80	52.8 ± 3.08	54.1 ± 2.18	49.5–58.6
Happiness	57.6 ± 3.48	60.1 ± 2.73	64.9 ± 3.08	60.9 ± 2.40	55.8–65.9

Note: Estimated Marginal Means for Each Emotion with 95% Confidence Intervals. SE = Standard Error; CI = Confidence Interval.

## Data Availability

The data presented in this study are available on request from the corresponding author due to ethical reasons.
